# Advanced Sandwich Composite Cores for Patient Support in Advanced Clinical Imaging and Oncology Treatment

**DOI:** 10.3390/ma13163549

**Published:** 2020-08-12

**Authors:** Robert H. Morris, Nicasio R. Geraldi, Lucy C. Pike, Jörg Pawelke, Aswin L. Hoffmann, Nicola Doy, Johanna L. Stafford, Abi Spicer, Michael I. Newton

**Affiliations:** 1 School of Science and Technology, Nottingham Trent University, Nottingham NG11 8NS, UK; nicasio.geraldi02@ntu.ac.uk (N.R.G.); mornic18@bilborough.ac.uk (N.D.); johanna.stafford2016@my.ntu.ac.uk (J.L.S.); Abi.Spicer02@ntu.ac.uk (A.S.); michael.newton@ntu.ac.uk (M.I.N.); 2 King’s College London and Guy’s and St Thomas’ PET Centre, School of Biomedical Engineering & Imaging Sciences, King’s College London, King’s Health Partners, St Thomas’ Hospital, London SE1 7EH, UK; lucy.pike@kcl.ac.uk; 3 OncoRay—National Center for Radiation Research in Oncology, Faculty of Medicine and University Hospital Carl Gustav Carus, Technische Universität Dresden, Helmholtz-Zentrum Dresden-Rossendorf, 01307 Dresden, Germany; Joerg.Pawelke@oncoray.de (J.P.); Aswin.Hoffmann@uniklinikum-dresden.de (A.L.H.); 4 Helmholtz-Zentrum Dresden-Rossendorf, Institute of Radiooncology-OncoRay, 01328 Dresden, Germany

**Keywords:** composite, core, clinical imaging, PET, CT, MRI, proton

## Abstract

Ongoing advances in both imaging and treatment for oncology purposes have seen a significant rise in the use of not only the individual imaging modalities, but also their combination in single systems such as Positron Emission Tomography combined with Computed Tomography (PET–CT) and PET–MRI (Magnetic Resonance Imaging) when planning for advanced oncology treatment, the most demanding of which is proton therapy. This has identified issues in the availability of suitable materials upon which to support the patient undergoing imaging and treatment owing to the differing requirements for each of the techniques. Sandwich composites are often selected to solve this issue but there is little information regarding optimum materials for their cores. In this paper, we presented a range of materials which are suitable for such purposes and evaluated the performance for use in terms of PET signal attenuation, proton beam stopping, MRI signal shading and X-Ray CT visibility. We found that Extruded Polystyrene offers the best compromise for patient support and positioning structures across all modalities tested, allowing for significant savings in treatment planning time and delivering more efficient treatment with lower margins.

## 1. Introduction

In order to drive ever greater outcomes from oncology treatment, technological advances are being continuously made to deliver new therapies and better instruments. One of the most recent advances is the use of proton therapy, where protons replace photons in traditional radiotherapy. This has seen rapid growth from just 14 systems globally in 2004 to 104 now operational, a further 38 in installation and commissioning and 28 more in the planning stage, thanks to highly improved patient outcomes [1]. This number is still significantly less than for traditional radiotherapy systems owing to the significant investment which they represent. Treatment is only half of the picture with the oncology workflow, with imaging being essential both to determine the best course of treatment but also to plan the shape and location of the treatment beams in the case of intensity modulated therapies such as Intensity Modulated Radio-Therapy (IMRT) and Intensity Modulated Proton-Therapy (IMPT) [2]. This imaging has traditionally been X-Ray Computed Tomography (CT) but over the past few decades has been slowly replaced by co-registered images from Magnetic Resonance Imaging (MRI) and CT owing to the superior soft tissue contrast which MRI brings. In the last few years, however, this has begun to be augmented with Positron Emission Tomography combined with CT (PET–CT) which has the ability to differentiate between cancerous and benign regions of neoplasia as well as modifying plans during treatment in so called response adapted therapy [3], thus, greatly reducing unnecessary treatment areas. The plethora of techniques which are now to be expected in a typical diagnosis, treatment planning and therapy cycle have uncovered a range of previously unforeseen challenges. In the early days of the inclusion of MRI in the workflow, it was the case that there was significant geometric distortion from a wide range of sources which are now routinely correct for in most vendor’s platforms [4]. However, it is still critical for optimum efficiency in the treatment planning and to minimise unnecessarily large treatment margins that the patient be in as close to the exact same position as possible for all imaging and treatment. This has led to the creation of a number of flat top couches for MRI and CT systems which are similar to those used in Radiotherapy systems. The routine nature now of such imaging protocols has seen the development of a range of new integrated systems which combine modalities to minimise the movement of patients between systems and to streamline their journey through diagnosis and planning to treatment. Examples of these include PET–CT, PET–MRI, MRI Linacs (Linear accelerators which deliver MRI guided photon therapy) and the like. This has however introduced new demands on the material requirements from which the patient flat couches are produced. Carbon fibre is ubiquitous in X-Ray modalities and is regularly used with PET. It is however well known to present significant image distortions and risks Eddy current induced heating which may harm the patient if it is used in MRI. In this work, we look to extend our knowledge of materials which are compatible with each of the modalities of interest such that it can be used to inform the best choice of materials from which to make patient support devices which can be universally applied between systems with minimal impact on the diagnostic or treatment power. The compatibility of composite skins and sandwich structures has been discussed elsewhere in the literature [5,6], but little information is available on the structural cores of sandwich composites which are typically used. Here, we employed a range of testing methods to evaluate the performance of four candidate core materials and concluded on their suitability for multimodality composite creation. 

## 2. Materials and Methods

The candidate cores suitable for use in advanced imaging and treatment modalities are tested for suitability in experiment on a proton therapy system and, MRI and PET–CT imaging systems. The methods applied to collect and process the data are presented in the following subsections after a brief outline of the samples under test.

### 2.1. Test Samples

Four core materials were chosen for evaluation based on materials which are commonly used in medical composites and those which are used in other areas but not found in a clinical setting. The materials must be low density, have high flexural modulus and be compatible with standard sandwich composite production methods including vacuum infusion which requires stability under vacuum. Two different foams are selected along with two different grain orientations of balsa wood as these see extensive use in sandwich composites in other sectors. Each material is cut to size according to the test which is to be performed, as detailed in the following sections. The materials are detailed in Table 1.

### 2.2. Proton Measurements

**Figure 1:**
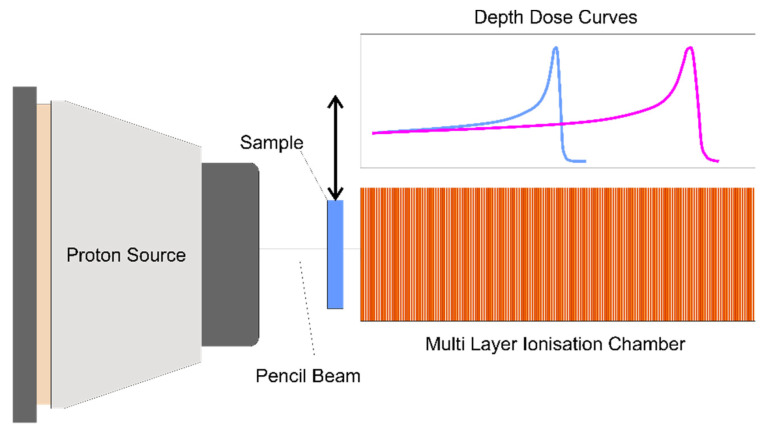
Setup of the proton experiment showing the arrangement for measurements. The depth dose curve is collected with the sample in and out of the beam.

The equivalent thickness of water required to reduce the proton beam energy to a given energy level is commonly referred to as the water equivalent thickness, or WET. The lower the WET value, the less stopping power of a given material. By determining the difference between proton range in water in the presence and absence of a material in the beam, the material specific WET (i.e., a measure by which the material slows down the beam) can be calculated. A more practical measure for comparison of materials is the proton stopping-power ratio or SPR which quantifies the change in proton range, normalised by thickness. Since this allows for comparison of materials, the SPR is used throughout this work. Range measurements were made at the University Proton Therapy Dresden, Germany, with monoenergetic pencil beams at three different energies: 100, 150 and 200 MeV. Depth dose curves were acquired using a multi-layer ionization chamber (MLIC) consisting of 180 stacked large electrodes of 12 cm diameter (Giraffe, IBA Dosimetry, Schwarzenbruck, Germany). The experimental setup is shown in Figure 1. The distal range of 80% of maximum dose (R80) was compared. The MLIC was previously calibrated to depth in water, i.e., the WET of an ionization chamber of the MLIC was determined. This was achieved by acquiring depth-dose curves of proton pencil beams with nominal energies ranging from 100 to 225 MeV by the MLIC and in-depth scanning a Bragg-peak ionization chamber (BPC, TM34070-2,5, PTW-Freiburg, Freiburg, Germany) in a water-filled phantom (Blue Phantom, IBA Dosimetry, Schwarzenbruck, Germany), for details see Supplement EB in [7]. The electrode plates have a 2 mm spacing providing for relative range measurements (WET determination) after MLIC calibration with an overall uncertainty of <0.1 mm [7]. Samples are sized at 10 cm × 10 cm for the measurements. 

The mean WET as a function of beam energy for several repeats is determined and range bars produced which are both plotted as a percentage deviation from the R80 in the absence of a sample, normalised by material thickness, yielding in the stopping-power ratio to water (SPR). The maximal measurement uncertainty was dominated by the thickness of the separate homogeneous slabs and experimentally determined as 2% related to the SPR. No dependence on beam energy was found for the measurements, a comparison of which can be found in supplementary information Figure S1. 

### 2.3. PET and CT Measurements

**Figure 2:**
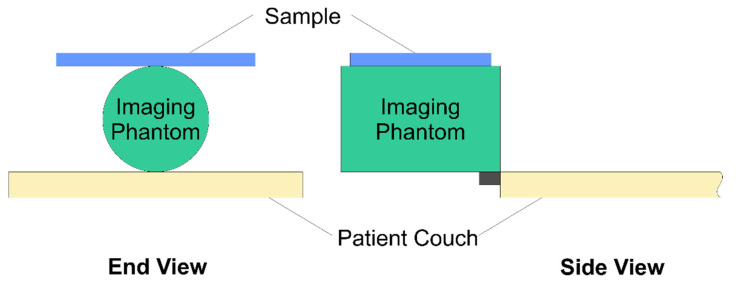
Setup for the Positron Emission Tomography combined with Computed Tomography (PET–CT) experiment showing the arrangement for measurements. Two views are shown of the setup for clarity.

A homogenous 63MBq Germanium-68 PET cylinder source (Eckert & Ziegler Isotope Products Inc., Valencia, CA, USA) was suspended in air off the end of the patient couch of a clinical PET–CT system (Biograph mCT Flow, Siemens Healthcare, Erlangen, Germany) using the daily quality control mount. Pieces of the material of interest, cut to 53 cm × 53 cm were held above this with a temporary fixing such that it could be easily changed between scans whilst keeping a fixed geometry. The experimental setup is shown in Figure 2. All acquisitions consisted of a CT scan followed by a single static PET acquisition centred over the 68-Ge cylinder. The PET acquisitions were set to acquire for 60 million true counts (defined with a window energy range of 435 to 650keV and coincidence window width of 4 ns). The CT images used for attenuation correction of the PET data were all acquired at 120 keV. Attenuation corrected PET images were reconstructed using the routine clinical parameters including time-of-flight data (2 iterations, 21 subsets, 5 mm Gaussian post filter). A final acquisition was performed using identical parameters without the material in place as a reference. It should be noted that the PET source is not collimated thus, although the geometry is matched for all experiments, any scattering caused by the material under test will be included in the count rate giving marginally reduced attenuation values. It is highly unlikely that this will have any great impact on the results since the materials being used are unlikely to result in significant scattering. 

The attenuation corrected PET images acquired with each material were compared to the reference PET images with no material by drawing a 18 cm region of interest in the centre of the phantom and calculating the standard deviation of the division. Since the corrected images would be used in practice, the in-built CT attenuation correction is verified to be effective by comparing these values between materials under test. Variations would indicate a mismatch between the scanner lookup table to convert between 120 kV CT and 511 keV attenuation and the actual linear attenuation of the materials under test [8].

To measure the attenuation of the 511 keV coincident photons caused by the presence of a support material the raw PET data prior to any attenuation correction was used. The raw sinogram output of the system contains an uncorrected true counts per second which is calculated and stored after every two seconds of data acquisition. The attenuation is then calculated as the difference between count rate in the presence and absence of the material under test divided by the count rate in its absence. The uncertainty on the measurements is determined by repeating the same procedure for the maximum and minimum counts per second stored in each of the raw measurement files. An attenuation of 100% represents no measured photons, whilst 0% attenuation represents no change in the measurement when compared to the phantom alone.

The influence of the material under test on the X-Ray CT imaging is assessed by firstly dividing each image by a reference image taken in the absence of any material such that any significant deviation from a value of 1 is due to the presence of alternative material. Areas which are greater than a value of 1.9 (positive and negative) are thresholded. Any regions which are less than 500 pixels in area are discarded leaving only the material. This image is used as a mask on the original data set, the value within which are averaged, and the standard deviation measured. These are then used to determine the percentage away from an ideal material (−1000 in Hounsfield Units, HU, as used in CT imaging), thus −1000 gives 0%, a material with the density of water would give 100% (0 in HU) and a material with the density of bone would give 200% (1000 in HU). In this way, it is possible to compare results of all imaging and treatment modalities on a single plot. As with the proton results, range bars are used based on the variation 

### 2.4. MRI Measurements

**Figure 3:**
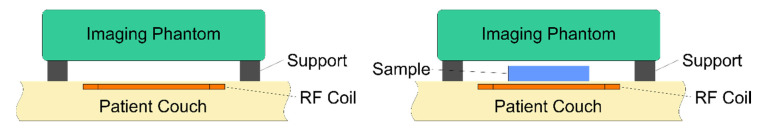
Setup for the Magnetic Resonance Imaging (MRI) experiment showing the arrangement for measurements. The left-hand image shows the without sample measurement, whilst the right-hand image is for measurements with sample.

Image of a wide flat phantom placed above 5 cm × 10 cm pieces of each sample are acquired using an MRI scanner (Avanto 1.5T, Siemens, Munich, Germany) running a standard turbo spin echo sequence and a gradient echo field mapping sequence, which is particularly sensitive to distortion and artefacts. A schematic of the measurement setup is shown in Figure 3. The phantom is filled with an aqueous solution of Nickel Chloride and Sodium Chloride as used in the American College of Radiology (ACR) phantom [9] to mimic the conductivity and relaxation rates of human tissue. The signal from images collected with and without samples (but in otherwise identical position) is divided to remove any influence of Radio Frequency (RF) coil inhomogeneity. A signal 5% above or below 1 indicates deterioration of the signal. 

### 2.5. Evaluation of Structural Properties

In order to normalise the resulting attenuation results, the flexural modulus of the cores is determined using an automated testing rig (6000R, Lloyd Instruments, Hampshire, UK) with 100 mm × 10 mm samples in compression mode with a 5 kN head (DLC, Lloyd Instruments, Hampshire, UK) using a standardised 3-point bend fixture. An example of the resulting data is shown in Figure S2 within Supplementary Material. The Flexural modulus *E_f_* is calculated according to Equation (1) [10].

(1) $$E_{f}=\frac{L^{3}m}{4bd^{3}}$$

where *b*, *d*, *L* and m are the width and thickness of the piece, the support span (60 mm with 10 mm diameter cylindrical points) and the gradient of the linear portion of the deflection curve respectively (which is shown in Supplementary Figure S2). The higher the flexural modulus, the stiffer the material. It is not possible to completely eliminate compression of the foam during the initial excursion of the test fixture. The mode of failure however is well represented by such an action since a crease ultimately forms due to skin buking which imparts the same compressive force. To allow for a full consideration of optimum materials the compressional modulus of the core materials is also measured using the same testing rig with a compressional test fixture. In this setup a rectangular head is driven into the material and the force as a function of head extension is measured. The compressive modulus E_C_ is defined as the ratio of stress and strain and is therefore calculated according to Equation (2).

**Figure 4:**
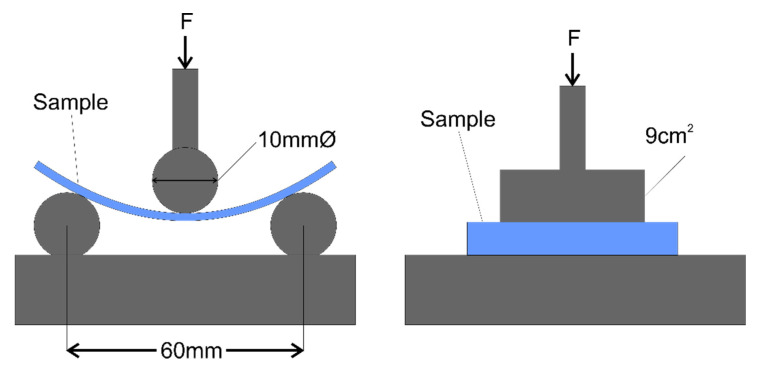
Setup for the three point bend test (**left**) and compressional modulus test (**right**).

(2) Ec=FAΔLL

where *F* is the applied force over a cross sectional area *A* (fixed at 9 cm^2^), Δ*L* is the extension and *L* is the sample thickness. Schematics of these two tests are shown in Figure 4. 

## 3. Results and Discussion

**Figure 5:**
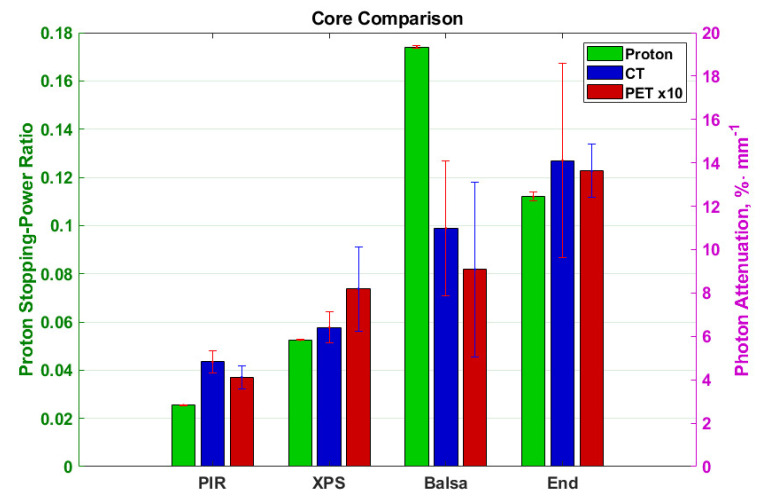
Thickness normalised photon attenuation and proton stopping-power ratio of materials in different modalities. Left to right are PolyIsocyanuRate (PIR), Extruded Polystyrene (XPS), along-grain balsa and end-grain balsa. Colours code different modalities as per the legend. The proton data is represented by the left y axis whilst the photon data (PET and CT) is represented by the right y axis. The PET data has been scaled to be 10-times larger to allow it to be visualised on the same axes. The errorbars for the PET and proton data are range bars (maximum value in data to minimum value in data) whilst the CT errorbars are 2 population standard deviations in length.

The photon attenuation normalised to thickness (i.e., divided by thickness) and proton stopping-power ratio (SPR) of the core materials are shown in Figure 5. It can be seen that there is a general upward trend with PIR being the least photon attenuating (0.4% mm^−1^ and 5% mm^−1^ for the PET and CT respectively) and proton stopping (0.025), and along-grain balsa or end-grain balsa being the most photon attenuating and proton stopping with peak values seven-times higher for balsa proton stopping-power ratio, 2.8-times higher for end-grain balsa in CT and 3.4-times higher for end-grain balsa in PET. The maximum difference in the material specific SPR for the proton measurements as a function of energy was 1.5% and shows no significant trend. The results are shown in Supplementary Material S1 for completeness.

**Figure 6:**
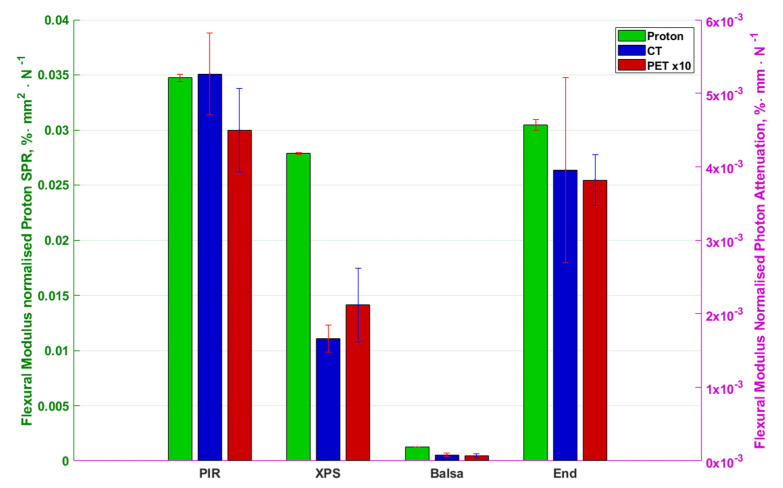
Flexural Modulus normalised photon attenuation and proton stopping-power ratio of materials in different modalities. Left to right are PIR, XPS, along-grain balsa and end-grain balsa. Colours code different modalities as per the legend. Proton stopping-power ratio is represented by the left right axis and CT and PET is represented by the right y axis. PET attenuation has been scaled to be 10× larger to allow for comparison on the same axes. The errorbars for the PET and proton data are range bars (maximum value in data to minimum value in data) whilst the CT errorbars are 2 population standard deviations in length.

**Figure 7:**
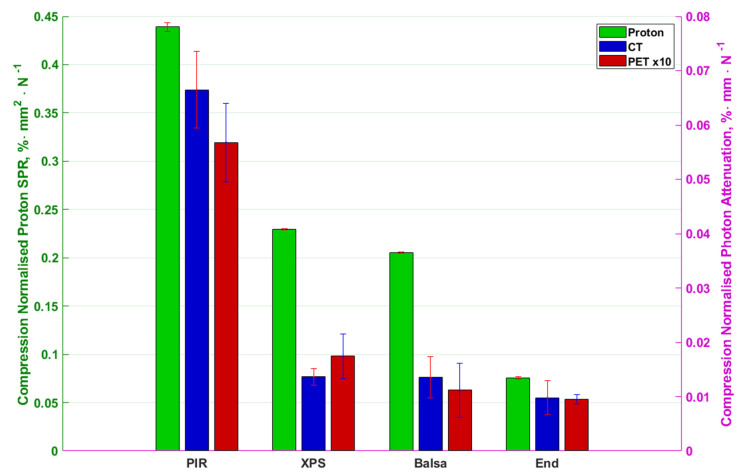
Compressional Stiffness normalised photon attenuation and proton stopping-power ratio of materials in different modalities. Left to right are PIR, XPS, along-grain balsa and end-grain balsa. Colours code different modalities as per the legend. Proton stopping-power ratio is represented by the left y axis while photon attenuation (PET and CT) are represented by the right y axis. The errorbars for the PET and proton data are range bars (maximum value in data to minimum value in data) whilst the CT errorbars are 2 population standard deviations in length.

Since the purpose of this research is to improve the production of patient support and positioning surfaces, the results are further normalised not by thickness but by flexural modulus in Figure 6 and compressional stiffness in Figure 7. This provides an indication of the attenuation and stopping to be expected to achieve a given physical property of material or product. 

The results in Figure 6 show along-grain balsa as the optimum material for such a support with values approximately 50-times smaller than the previously best performing PIR. Figure 7 on the other hand shows end-grain balsa to be the least photon attenuating and proton stopping per unit compressional stiffness with approximately 7-times improvement over PIR for all modalities. The errorbars overlap for the end-grain and along-grain balsa suggesting similar performance.

**Figure 8:**
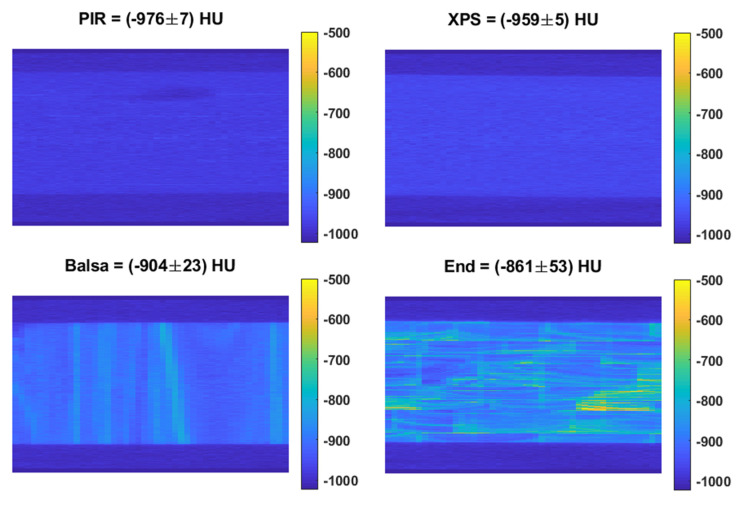
Planar image homogeneity for materials in X-Ray CT. The title for each image shows the mean ± standard deviation within the sample region.

Figure 8 shows the planar homogeneity of each of the materials for X-Ray CT. It can be seen that the along-grain balsa and end-grain balsa have significant inhomogeneity with standard deviations 3.3-and 8.8-times higher (respectively) than the PIR and XPS materials. This may affect diagnostic potential in planar imaging modalities, which are key to many radiotherapy treatment sessions, where this variation will be merged indiscriminately with anatomical features. 

The MRI results are not shown since it was found that none of the materials produced a measurable attenuation above the level of the noise but are included in Figure S3 of Supplementary Materials for completeness. Similarly, the verification of performance for the PET normalised images are shown in Figure S4 since there is no variation.

When considering only the photon attenuation of the various modalities and proton stopping, the PIR performs the best, presenting the minimum attenuation in all tests. However, when normalising by flexural modulus the cross-grain balsa becomes the optimum choice with the XPS presenting more than 10-times greater attenuation and stopping across modalities. When normalised by compressional stiffness however the performance of the along-grain balsa is similar to that of the XPS across all modalities whilst the end-grain balsa presents the minimum attenuation and stopping. Unfortunately, the homogeneity of both grains of balsa is not sufficient for planar imaging where the whole coronal view is averaged as is commonly employed in megavoltage imaging prior to radiotherapy. As such, the choice of core material will depend on the application to maintain diagnostic potential. Where a multimodal material is desired, XPS should be used. Where no coronal X-ray imaging is intended, along grain or end-grain balsa represent the most suitable material choices. Where a tough skin is applied in a composite the along-grain balsa offers the better choice thanks to the lower photon attenuation and proton stopping normalised to flexural modulus, whilst end-grain balsa with minimal attenuation and stopping normalised to compressional stiffness will be the better choice in applications where core damage is possible through thin or weak skins. It should be noted that the highest signal intensity on the two foams is not visible in any of the standard CT windows where the lowest HU value visible is typically around −800 [11]. In the case of the balsa, however, it is possible that some striation will be visible.

In this article we have presented measurements of the core materials alone to allow for the reader to use this as the basis of an informed choice for construction of patient positioning substrates. It should be noted however, that any such choice should be verified with similar measurements to those presented here with the final sandwich composite structure, including skins to ensure maximum performance.

## 4. Conclusions

From the four materials tested for use as a core in patient support structures where tomographic imaging is undertaken, there is no issue with the planar homogeneity. However, if the application involves planar imaging, for example Megavoltage imaging for radiotherapy, this inhomogeneity may have negative implications for diagnostic potential. It is unlikely that the HU values in the balsa will be visible in X-Ray CT imaging but the best compromise between flexural modulus, attenuation or stopping and visibility is seen across all modalities for the XPS. It should be noted however that the final product must be thicker for the same structural performance. 

## Figures and Tables

**Table 1:** Material information.

Material	Specifics	Formula	Molecular Weight (g/mol)	Density (kg/m^3^)	Source
PolyIsocyanuRate (PIR)	GA4000	(C3-H3-N3-O3)n	129.1	170–210	Celotex, Ipswich, UK
Extruded Polystyrene (XPS)	GG700	(C8-H8)n	104.1	45	Kingspan, Kingscourt, Ireland
Along-Grain Balsa		45% (C6-H10-O5)n 25% (C31-H34-O11)n 25% (C5-H10-O5)n	162.1 582.6 150.1	160	Hobbycraft, Dorset, UK
End-Grain Balsa		45% (C6-H10-O5)n 25% (C31-H34-O11)n 25% (C5-H10-O5)n	162.1 582.6 150.1	200	Allscot, Glasgow, UK

## Supplementary Materials

The following are available online at https://www.mdpi.com/1996-1944/13/16/3549/s1, Figure S1: Comparison of beam energy dependence on proton stopping-power ratio showing no significant relationship between beam energy and stopping-power ratio, Figure S2: Example force vs displacement plot demonstrating the fitting of the linear region, Figure S3: Influence of core materials on MRI signal. The top panel of images are phase maps for the phantom collected directly above the samples. The phase is adversely affected by both cuts of balsa although they do not induce any variation across the sample itself and are below the level which are expected to cause any variation in clinical images. Clinical images are normalised by the mean of three blanks and are shown in the middle bottom panel. The pattern of the images is owing to the motion of the fluid caused by convection. As can be seen none of the images display any significant variation outside of that seen on the two blank images. The lower panel of images shows the region of the clinical images collected directly over the samples. No variation is detectable at the normal windowing expected for a clinical image suggesting that all materials are suitable for use, Figure S4: Averaged Line profile over corrected PET images. Any mismatch in the lookup tables between 120kV CT and 511keV attenuation would present as a significant variation in the y axis of this plot. None can be seen suggesting that all of the core materials are suitable for use in PET imaging.
