# Supplementary material for: Advanced Sandwich Composite Cores for Patient Support in Advanced Clinical Imaging and Oncology Treatment
Source: Materials (Basel). 2020 Aug 12;13(16):3549. doi: 10.3390/ma13163549 (PMC7475909; doi:10.3390/ma13163549)
Supplement: Supplementary file 1 [file materials-13-03549-s001.pdf]

materials

MDPI

Communication

Advanced Sandwich Composite Cores for Patient Support in Advanced Clinical Imaging and Oncology Treatment

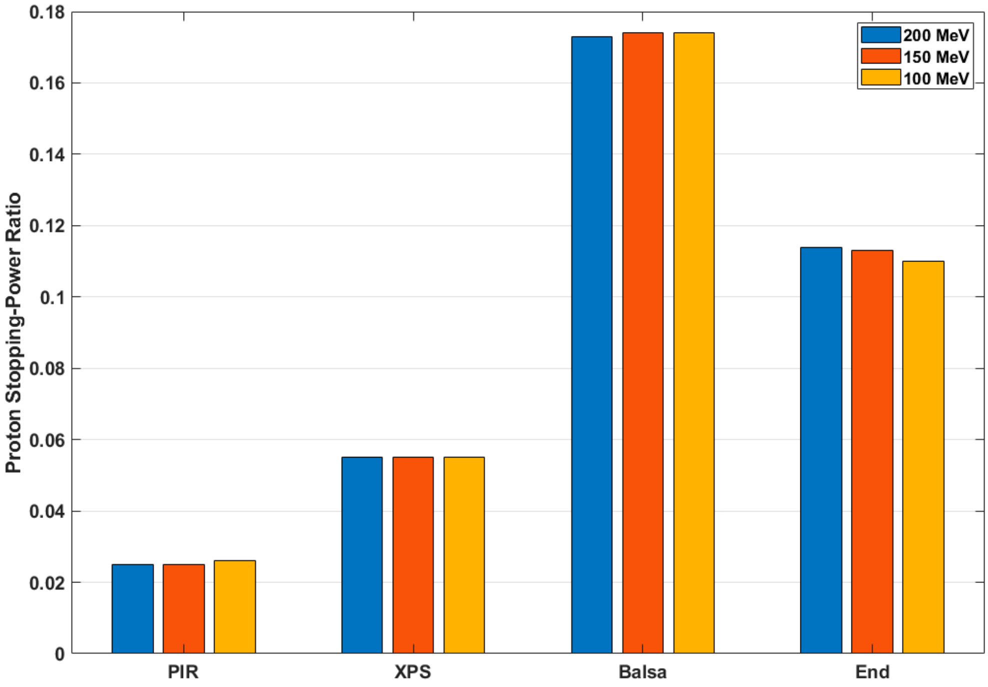

Figure S1. Comparison of beam energy dependence on proton stopping-power ratio showing no significant relationship between beam energy and stopping-power ratio.

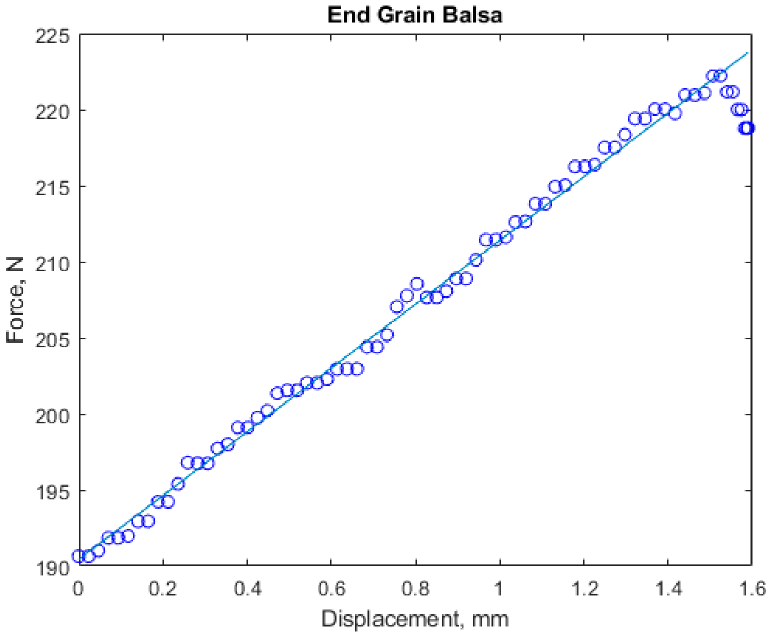

Figure S2. Example force vs displacement plot demonstrating the fitting of the linear region.

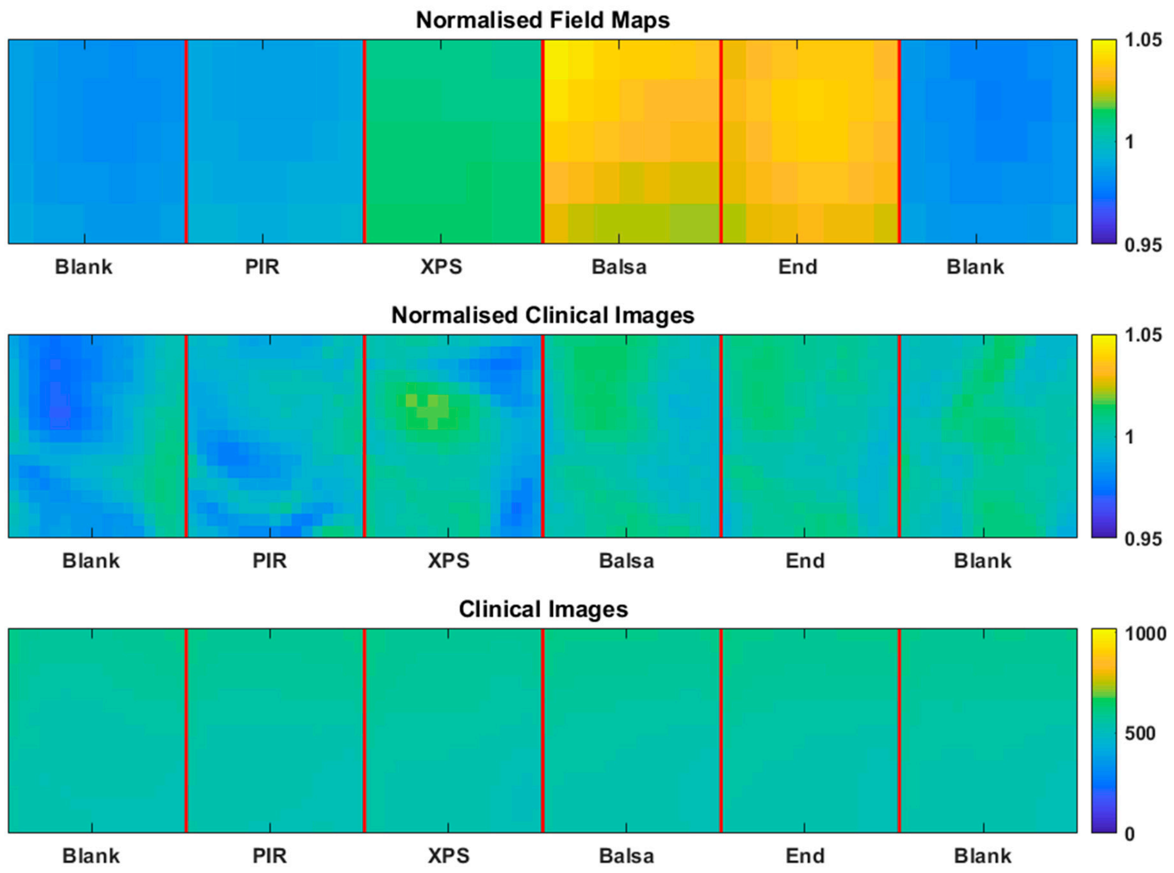

Figure S3. Influence of core materials on MRI signal. The top panel of images are phase maps for the phantom collected directly above the samples. The phase is adversely affected by both cuts of balsa although they do not induce any variation across the sample itself and are below the level which are expected to cause any variation in clinical images. Clinical images are normalised by the mean of three blanks and are shown in the middle bottom panel. The pattern of the images is owing to the motion of the fluid caused by convection. As can be seen none of the images display any significant variation outside of that seen on the two blank images. The lower panel of images shows the region of the clinical images collected directly over the samples. No variation is detectable at the normal windowing expected for a clinical image suggesting that all materials are suitable for use.

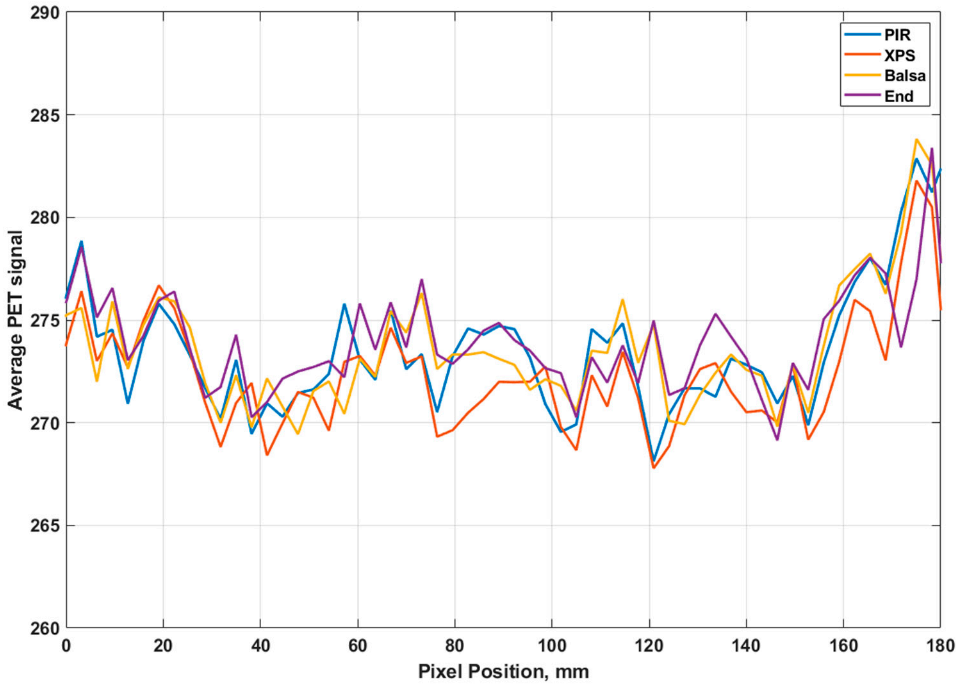

Figure S4. Averaged Line profile over corrected PET images. Any mismatch in the lookup tables between 120kV CT and 511keV attenuation would present as a significant variation in the y axis of this plot. None can be seen suggesting that all of the core materials are suitable for use in PET imaging.

© 2020 by the authors. Submitted for possible open access publication under the terms and conditions of the Creative Commons Attribution (CC BY) license (http://creativecommons.org/licenses/by/4.0/).
